# Bionomics of *Anopheles latens *in Kapit, Sarawak, Malaysian Borneo in relation to the transmission of zoonotic simian malaria parasite *Plasmodium knowlesi*

**DOI:** 10.1186/1475-2875-7-52

**Published:** 2008-03-31

**Authors:** Cheong H Tan, Indra Vythilingam, Asmad Matusop, Seng T Chan, Balbir Singh

**Affiliations:** 1 Infectious Diseases Research Centre, Institute for Medical Research Jalan Pahang, 50588 Kuala Lumpur, Malaysia; 2 Malaria Research Centre, University Malaysia Sarawak., Kuching, Sarawak, Malaysia; 3 Sarawak Department of Health, Kuching, Sarawak, Malaysia

## Abstract

**Background:**

A large focus of human infections with *Plasmodium knowlesi*, a simian parasite naturally found in long-tailed and pig-tailed macaques was discovered in the Kapit Division of Sarawak, Malaysian Borneo. A study was initiated to identify the vectors of malaria, to elucidate where transmission is taking place and to understand the bionomics of the vectors in Kapit.

**Methods:**

Three different ecological sites in the forest, farm and longhouse in the Kapit district were selected for the study. Mosquitoes were collected by human landing collection at all sites and at the forest also by monkey-baited-traps situated on three different levels. All mosquitoes were identified and salivary glands and midguts of anopheline mosquitoes were dissected to determine the presence of malaria parasites.

**Results and Discussions:**

Over an 11-month period, a total of 2,504 *Anopheles *mosquitoes comprising 12 species were caught; 1,035 at the farm, 774 at the forest and 425 at the longhouse. *Anopheles latens *(62.3%) and *Anopheles watsonii *(30.6%) were the predominant species caught in the forested ecotypes, while in the farm *Anopheles donaldi *(49.9%) and *An. latens *(35.6%) predominated. In the long house, *An. latens *(29.6%) and *An. donaldi *(22.8%) were the major Anopheline species. However, *An. latens *was the only mosquito positive for sporozoites and it was found to be attracted to both human and monkey hosts. In monkey-baited net traps, it preferred to bite monkeys at the canopy level than at ground level. *An. latens *was found biting early as 18.00 hours.

**Conclusion:**

*Anopheles latens *is the main vector for *P*. *knowlesi *malaria parasites in the Kapit District of Sarawak, Malaysian Borneo. The study underscores the relationship between ecology, abundance and bionomics of anopheline fauna. The simio-anthropophagic and acrodendrophilic behaviour of *An. latens *makes it an efficient vector for the transmission of *P. knowlesi *parasites to both human and monkey hosts.

## Background

Malaria parasites in Peninsular Malaysian monkeys were first reported in 1908 [1], but only gained prominence in the 1960's after the accidental discovery [2] that *Plasmodium cynomolgi *could be transmitted to humans via mosquito bites in the laboratory. This stimulated interest at a time when the Malaria Eradication Programme was initiated by the World Health Organization [3] and it was important to determine if malaria was a zoonosis. Therefore, extensive studies were carried out in Peninsular Malaysia to determine the distribution, prevalence and species of malaria parasites in monkeys and apes and the natural vectors of monkey malaria parasites [4-7]. Instead of uncovering human cynomolgi malaria infections, *Plasmodium knowlesi *was the first simian malaria parasite found to be infecting humans in nature. The first case was reported in 1965 from the state of Pahang [8], Peninsular Malaysia, followed by a second case five years later acquired from Johore, Peninsular Malaysia [9]. It was postulated that *P. knowlesi *could be transmitted from monkeys to man and laboratory studies proved that it was possible [10]. However, a large scale study that was initiated in Pahang to investigate whether malaria was a zoonosis, by a group of American and local researchers based at the Institute for Medical Research in Kuala Lumpur, Peninsular Malaysia, concluded that simian malaria in humans was an extremely rare event [11,12]. This was based on their studies in which they collected blood samples from more than 1,100 local residents, pooled the samples and injected them into rhesus monkeys and none of the monkeys contracted malaria. However, in 2004 a large focus of human *P. knowlesi *infection was reported in the Kapit Division of Sarawak [13]. In that study 71.6% (101/141) of human malaria cases at Kapit Hospital which had been identified by microscopy as single *Plasmodium malariae *infections were actually *P. knowlesi *and other non-*P. malariae *species by nested polymerase chain reaction (PCR) assays. *Plasmodium knowlesi *is naturally found in long-tailed macaques (*Macaca fascicularis*), pig-tailed macaques (*Macaca nemestrina*) [14] and banded leaf monkeys (*Presbytis malalophos*) [15,16]. Since transmission of this zoonotic parasite to humans is occurring in the Kapit Division of Sarawak, Malaysian Borneo, it is important to identify the vectors so that appropriate measures can be planned and initiated to control the spread of simian malaria in humans.

Numerous studies on vectors of human malaria have been carried out in Sarawak [17-20] but none of these have been undertaken in the Kapit District of Sarawak. Several anopheline species incriminated or suspected to transmit monkey malaria parasites in Peninsular Malaysia, such as *Anopheles latens*, *Anopheles balabacensis *are also present in Sarawak [21]. Hence, the vectorial status of the anopheline species present in the Kapit Division needs to be ascertained in order to determine if they are competent vectors of simian malaria parasites. Furthermore, the epidemiological data of *P. knowlesi *infection in humans revealed that infections occur primarily in adults and no clustering of cases occurred within communities that live in communal longhouses, which suggests transmission of *P. knowlesi *to humans occurred away from the vicinity of the longhouses [13].

Thus the objectives of this study were to determine the vectors of *P. knowlesi *and other simian malaria parasites in the Kapit Division of Sarawak; and to study the dynamics of these vectors in different ecological sites in order to elucidate the most likely place where transmission was taking place. Preliminary results of this study were reported where *An. latens was *incriminated as the vector for *P. knowlesi *[22]. Here detailed results of this eleven-month study on vectors of malaria and their bionomics in the Kapit district of Sarawak, Malaysian Borneo is presented.

## Methods

### Study sites

The study was carried out in Kapit district from June 2005 to April 2006. The district is part of Kapit division located in the central part of Sarawak, Malaysian Borneo. Kapit division is bordered by Kalimantan, Indonesia in the south and east, Miri and Bintulu divisions in the north and Sibu division in the west. The Kapit district is a hilly area covered by tropical rain forests. Three different ecological sites were selected based on human cases of *P*. *knowlesi *and or presence of long-tailed macaques (*M. fascicularis*). The first site was a longhouse (residence of the indigenous people) in Lubok Loh Yong situated 16 km from the town center and is close to the river and surrounded by trees and shrubs. One of the residents of the longhouse had been admitted to Kapit Hospital with knowlesi malaria in 2004. The second site was a farm in Ulu Sungai Yong on land cleared of original forest and now supporting fruit trees and secondary vegetation. There is a small hut in the farm and a stream is running through the farm. There is only one house situated just outside the farm. Pig-tailed macaques (*M. nemestrina*) are often sighted in this area and one of the farmers had previously been admitted to Kapit Hospital with knowlesi malaria. The distance between the longhouse and farm is 5 km. The third site was a forest 4.5 km away from Kapit town center and is 1,200 meters above sea level. The forest is only accessible by four wheel drive vehicle but during the rainy season it is only accessible by foot. Long-tailed macaques were often seen in this area, which is also approximately 1 km from the house of a malaria patient admitted to Kapit Hospital in 2001 with knowlesi malaria.

### Mosquito collections

All night mosquito collections, using the bare-leg catch method [23] were performed during the eleven-month study period. In each area, three nights of collections were carried out every month. In the longhouse both indoor and outdoor collections were carried out by two men each while in the farm and forest fringe only outdoor collections were performed by three men working in two shifts. The catches were performed from 18.00 to 06.00 hours.

### Monkey-baited-trap

In the forest, a comparison was made between the number of mosquitoes attracted to human bait on the ground and the numbers attracted to monkey bait at ground level, on platforms at three meters and six meters on the forest canopy. In the first two months only one monkey was placed in each cage but in subsequent months there were two monkeys per cage. The cages with the monkeys were placed on the platform inside a mosquito net measuring 190 cm × 180 cm × 150 cm with an opening of about 40 cm on either ends. The method used was similar to that used by Wharton [24]. Platforms were constructed among the branches of a tree. The traps were operated from 18.00 to 06.00 hours and were searched at two hourly intervals. A collector, upon entering the net, closed the opening and collected all resting mosquitoes with the use of a suction tube.

### Mosquito identification and dissection

All mosquitoes were identified morphologically in the field laboratory. The keys of Reid [21] were used for the identification of *Anopheles *mosquitoes. Generally most *Anopheles *mosquitoes were dissected to extract ovaries for parity determination and the midguts and salivary glands were examined for oocysts and sporozoites respectively. The remaining pair of the salivary glands of all parous anopheline mosquitoes were placed in an individual 1.5 ml microcentrifuge tubes (Axygen, USA) containing 95% alcohol and were labeled accordingly. In addition, all positive midguts were preserved using the same method. The preserved salivary glands and midguts were used for molecular studies [22].

### DNA extraction and PCR

Ethanol used to preserve the salivary glands and midguts of malaria positive mosquitoes were allowed to evaporate completely. To accelerate the evaporation, all 1.5 ml tubes containing the preserved specimens were incubated in a dry bath (Thermomixer, Eppendorf, Germany) set at 70°C. Genomic DNA of the dried salivary glands was extracted using DNeasy^® ^tissue Kit (Qiagen, Germany) and performed according to the manufacturer's protocol. The eluted DNA was kept at 4°C until required. A nested PCR assay [13] based on the *Plasmodium *DNA sequence of the small subunit ribosomal RNA (SSUrRNA) genes was used to detect and identify the species of malaria parasites found in *Anopheles *mosquitoes caught in Kapit.

### Ethical clearance

This project was approved by the Medical Research & Ethics Committee Ministry of Health Malaysia (KKM/JEPP/02(110). All volunteers who carried out mosquito collections were provided with antimalarial prophylaxis.

## Results

### Species composition and spatial distribution of Anopheles species collected in different ecological sites

A total of 2,504 *Anopheles *mosquitoes belonging to 12 species were caught biting humans and monkeys (Table 1). Of these, 10 species were obtained biting man while two (*Anopheles pujutensis*, *Anopheles macarthuri*) were caught exclusively biting monkeys. *Anopheles latens *comprised 42.9% of the total collection. Besides *An. latens*, the two predominant species were *Anopheles donaldi *in the farm and *Anopheles watsonii *in the forest.

**Table 1:** Mosquitoes collected from three different ecological sites in Kapit, Sarawak from June 2005-April 2006.

*Anopheles *species	Farm	Long House		Forest		Total (%)
		
		In	Out	BLC	MBT	
*An. barbirostris*	5	0	3	0	0	8 (0.3)
*An. donaldi*	651	35	62	2	1	751 (30)
*An. introlatus*	3	0	1	0	0	4 (0.2)
*An. latens*	465	37	89	273	209	1073 (42.8)
*An. macarthuri*	0	0	0	0	8	8 (0.3)
*An. maculatus*	6	0	1	11	2	20 (0.8)
An. pujutensis	0	0	0	0	9	9 (0.4)
*An. roperi*	0	1	2	0	0	3 (0.1)
*An. tesselatus*	9	15	38	0	0	62 (24.8)
*An. vanus*	84	25	68	2	3	182 (7.3)
*An. watsonii*	1	1	1	157	80	240 (9.6)
*An*. *kokhani*	81	19	27	15	2	144 (5.8)

Total	1305	133	292	460	314	2504

There were observed differences in species composition and abundance between different ecological sites. *Anopheles latens *(62.3%) and *An. watsonii *(30.6%) were the predominant species caught in the forested ecotype, while in the farming area *An. donaldi *(49.9%) and *An. latens *(35.6%) were predominant. In the long-house, *An. latens *(29.7%), *An. donaldi *(22.8%) and *Anopheles vanus *(21.9%) were found to be the major anopheline species caught, albeit the numbers of mosquitoes caught indoors were less as compared to the outdoor collection. *Anopheles kokhani*, a species new to science [25], was also observed in all three sites. The rest of the species were found in varying numbers depending on where they were collected.

### Changes in landing rate over time

**Figure 1:**
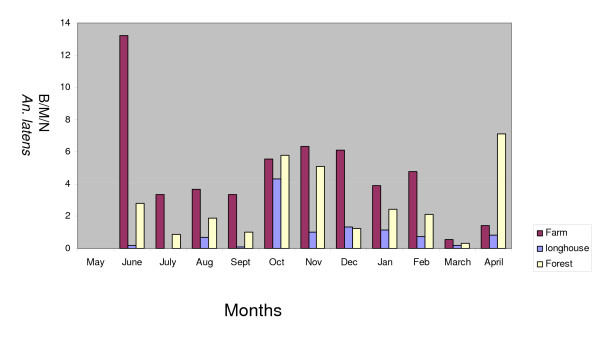
Vector density of *Anopheles latens *at the study sites.

The biting peak of *An. latens *in the farm was in June and from October to December 2005 and in February 2006, while in the forest there was an increase in biting rate in October and November 2005 and in April 2006 (Figure 1). However, in the longhouse, the peak was observed to be in October 2005. Studies in the past have shown that the amount of rainfall affects the anopheline biting density [26,27]. During the study period there was no meteorological station in Kapit and the nearest meteorological station was more than 100 km away in the Division of Sibu. Consequently, it was not possible to determine the effect of rainfall on the monthly densities of mosquitoes in this study.

### Biting cycles

**Figure 2:**
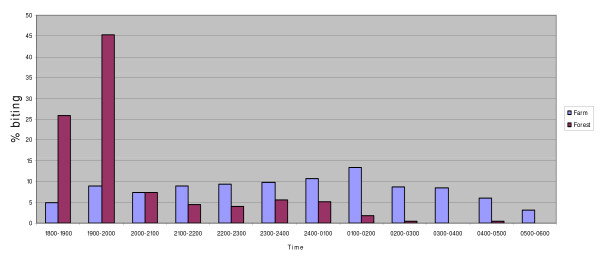
Biting times of *Anopheles latens *in farm and forest.

In the forest, *An. latens *comes to bite as early as 18.00 hours in the evening but the peak biting time is between 19.00 and 20.00 followed by a sharp decline thereafter as shown in Figure 2. While in the farm it starts to bite from 18.00 hours and continues to bite throughout the night until dawn (06.00 hours) and the peak biting time is between 01.00 to 02.00 hours. In the longhouse, *An. latens *showed a peak outdoor biting time between 23.00 h to 02.00 h while a peak biting time indoor between midnight and 02.00 h was observed.

### Monkey-baited trap

A total of 312 *Anopheles *mosquitoes were caught in the monkey-baited trap. Of these, *An. latens *was the predominant species followed by *An. watsonii*. The results of mosquitoes obtained from the monkey-baited traps are summarized in Table 2. *Anopheles latens *preferred to bite at six meters rather than three meters and ground level. The ratio of ground:3 m:6 m was as follows, 1:10.3:18.4. Most were found entering the trap between 18.00 to 21.00 hours. Based on results from this study, the *An. latens *biting ratio of monkey to human was 1:1.3.

**Table 2:** *Anopheles latens *obtained at the monkey-baited-traps

	21 hours	23 hours	0100	0300	0500	Total (%)
Ground	1	2	3	0	1	7 (3.3)
3 Meters	41	12	9	4	7	73 (34.9)
6 Meters	47	25	32	6	19	129 (61.7)

Total	89	38	44	10	27	209

### Parous rate, daily survival rate, life expectancy and vectorial capacity of *Anopheles latens*

The parous rates of *An. latens *and the confidence interval is shown in Table 3. Overall, more than 59% of the *An. latens *caught in Kapit were parous, however a significant difference was observed between the parous rates of *An. latens *caught in different ecological sites. The parity rate observed in *An. latens *caught at the farm was similar to those collected in the longhouse (65.8%). However, the parous rate of *An. latens *caught at the forest was observed to be significantly lower than those caught at the farm (X^2 ^= 13.27, P < 0.05) and the longhouse (X^2 ^= 5.67, P < 0.05).

**Table 3:** Parous rate, probability of survival, probability of transmission of malaria parasite, life expectancy and vectorial capacity of *An. latens*

	Farm	Forest	Longhouse
Parous rate (95% CI)	65.8 (61.2–70.1)	53.7 (48.9–58.3)	65.8 (57.0–73.9)
Probability of daily survival -p	0.87	0.81	0.87
p^10 ^(%)	25	13	25
Life expectancy P^10 ^/-log_e _p (days)	7.2	4.7	7.2
Vectorial capacity	2.86	0.60	0.85

*p*^10 ^percentage of population expected to live long enough to become infective with an extrinsic cycle of 10 days based on *P. knowlesi *extrinsic incubation period [32].

The daily survival rate [28], life expectancy [29] and vectorial capacity [30] of *An. latens *are shown in Table 3. Overall life expectancy of *An. latens *was highest in the farm and longhouse and followed by those found in the forest. In the farm and long-house 25% of *An. latens *would be expected to live the 10 days necessary for the *P. knowlesi *sporozoites to be formed. Those surviving the 10 days would have a further life expectancy of 7.2 days. For this calculation results of mosquitoes caught outdoors were pooled together with the results of those caught inside the long-house. The vectorial capacity of the *An. latens *was found to be highest in the farm, and lowest in the forest.

### Sporozoite rate and annual entomological inoculation rates

A total of nine *An. latens *were found to harbour the sporozoite stage of malaria parasites and all were of simian origin. Of these, six were caught in the forest, and the rest in the farm area. Two of the infective *An. latens*, caught in the forest, were found to be positive for *P. knowlesi *[22], including one that was caught in the monkey-baited net trap. In addition, two *An. latens *caught attracted to humans in the farm, were also observed to harbour the sporozoite stage of *P. knowlesi *[22]. The remaining five infective *An. latens *were found to carry other species of simian malaria parasites. The overall sporozoite rates in both the farm and the forest were 0.7% and 1.4%, respectively (Table 4). The computed annual entomological inoculation rates (EIR) were 11.98 and 14.10 in the farm and in the forest, respectively. However, the annual EIR for *P. knowlesi *was 7.8 and 4.6 in the farm and forest respectively and this in not statistically significant (X^2 ^= 0.22 P > 0.05).

**Table 4:** Sporozoite and Oocyst rate of positive mosquitoes in study sites

Mosquito Species	Number dissected		Sporozoite Rate (95% CI)		Oocyst Rate (95% CI)	
	
	Farm	Forest	Farm	Forest	Farm	Forest
*An. latens*	435	438	0.69 (0.18–2.18)	1.37* (0.56–3.11)	0.46 (0.08–1.84)	1.37 (0.56–3.11)
*An. watsonii*	1	224	0	0	0	3.13 (1.38–6.60)
*An. donaldi*	641	1	0	0	0.16 (0.01–1.00)	0

* One of the infective *An. latens *was caught in the monkey baited net trap

There were five *An. latens*, seven *An. watsonii *and one *An. donaldi *that were positive for the oocyst stage (Table 4). In addition, three of the infective *An. latens*, that were caught in the forests, also had oocysts in their midgut (Table 4). None of the mosquitoes collected from the longhouse had malaria parasites

## Discussion

In the 1960's, it was suggested that mosquitoes of the *Anopheles leucosphyrus *group, may provide a link between humans and monkeys and that if simian malaria is transmitted to man in nature, it is likely to be in areas where these mosquitoes are common [31]. This has been confirmed by the current study where *An. latens *has been incriminated as the vector of *P. knowlesi *in nature in the Kapit division [22] where a large focus of naturally acquired *P. knowlesi *infection in humans was reported [13]. Aside from being the most predominant anopheline mosquitoes caught biting humans in Kapit, *An. latens *was observed to be attracted to both human and monkey hosts. In the monkey-baited net traps, it was also observed that a significant number of *An. latens *were collected at the higher levels compared to those caught at ground level showing an acrodendrophilic behaviour. This is important as the natural monkey host is arboreal in nature. In order for this malaria parasite to be maintained in nature and for transmission to man to occur, the vector needs to be highly simio-anthropophagic in nature. If the vector for *P. knowlesi *is highly simiophagic, as in the case of *Anopheles hackeri *in Peninsular Malaysia [4], transmission in the natural hosts may be maintained, but transmission to humans will be rare [32].

The fact that *An. latens *is attracted to monkeys in the canopy and humans on the ground indicates that both humans and monkeys could be exposed to infection from each other. Data from this study area indicate that the zoonotic parasite, *P. knowlesi*, is being transmitted to both humans and macaques by *An. latens*. Thus, in Kapit, humans get the infection when their forest associated activities, such as farming, logging, or hunting exposes them to *An. latens*. Throughout the course of this study infected mosquitoes were not obtained from the longhouse and the number of anophelines caught there were also small.

Like all members of the Leucosphyrus group, *An. latens *is mainly a forest breeding mosquito associated with dense jungle and forest fringes [21,33]. In the current study, nearly 90% of the total *An. latens *were collected at either the farm (40%) or the forest (50%) and were consistent with earlier studies conducted in other areas of Sarawak. In addition, only 126 *An. latens *(10%) were collected in the longhouse, and of these 71% were collected outdoors. Entomological studies conducted in other areas in Sarawak [18,19] showed that *An. latens *were commonly found in farming zones that were located at the forest fringe rather than in villages, and the density of *An. latens *decreases in relation to distance away from the jungle.

It is known that multiple factors determine the prevalence of malaria and one of these is the intensity of malaria transmission which is defined as the rate at which people get inoculated with malaria parasites from mosquitoes [34]. In the current study, only *An. latens *were found to be infective, and the average number of infectious bites by these mosquitoes in both the farm and the forest during the entire duration of the study was 11.98 and 14.1 per year respectively, which is higher compared to the rates in many Asian countries [34]. When considering the risk of acquiring *P. knowlesi *in Kapit, the intensity of transmission has been shown to be comparable for both ecotypes, hence the risk of acquiring *P. knowlesi *in either the forest or the farm is the same. In Sarawak, the forests and their surrounding areas are recognized focal points for malaria transmission whenever *An. latens *is present in large numbers [20].

Although *An. latens *has been incriminated as the vector of *P. knowlesi *in Kapit, the role of other anopheline species as a possible vector for *P. knowlesi *in Kapit needs further assessment. This is especially true for *An. pujutensis*, *An. introlatus*, and *An. macarthuri*, all of which were caught in small numbers and were not dissected. They have been reported to be simiophagic and have either been suspected or been incriminated as vectors of simian malaria parasites in other localities in Peninsular Malaysia [32,35,36]. To determine if they are vectors of *P. knowlesi*, intensive entomological surveys have to be carried out in many parts of the forest and in other parts of the Kapit Division. With the advancement of molecular techniques it is possible to identify the sporozoites to species level and thus vectorial status can be determined.

*Anopheles watsonii*, a forest species, was caught in large numbers, but only in the forest, where it was found to be attracted to both human and monkey hosts. Previously, a small numbers of this species were also obtained in hill forest in Peninsular Malaysia at night with human and monkey- baited traps [35]. They obtained more mosquitoes in the canopy than at ground level, but all were negative by dissection. Thus earlier workers did not consider *An. watsonii *as a vector of human malaria. In the current study, oocysts were found by dissection and the numbers in each gut ranged from 7–90. However, none were positive for sporozoites by dissection. Since they are attracted to humans and non human primates, further studies should be conducted on this species before any definitive conclusions can be inferred about their vectorial status.

With large tracts of forest being cut down, non-human primates are coming close to the human environment. Human cases of *P. knowlesi *have been reported not only in Sarawak[13], but also in Thailand [37], Manymar [38] and in Peninsular Malaysia [8,9,39]. Thus control strategies for malaria in Southeast Asia may have to change to include this zoonotic aspect of the transmission.

## Conclusion

This study has shown that *An. latens*, previously incriminated as the main vector for *P*. *knowlesi *in Kapit District of Sarawak, Malaysian Borneo has the highest vectorial capacity among all Anopheline species caught in the area. The abundance and composition of anopheline species was found to be dependent on the ecotype studied, with *An. latens *being predominantly found in the forest and farm. The simio-anthropophagic and acrodendrophilic behaviour of *An. latens *has been established. These evidence-based findings will be useful for the planning of control strategies for malaria vectors.

## Competing interests

The author(s) declare that they have no competing interests.

## Authors' contributions

IV and BS conceived the study. IV, CHT, BS were responsible for preparation of manuscript. IV, CHT, STC and AM were responsible for field collection, supervision, identification and processing of mosquitoes. BS obtained permits to collect and undertake research on biological specimens from Sarawak Biodiversity Council and permits to trap and keep monkeys in captivity from Sarawak Forestry Department. CHT conducted molecular work. All authors have read and approved the manuscript.

## References

[B1] Daniels CW (1908). Animal parasites in man and some of the lower animals in Malaya. Stud Inst Med Res FMS.

[B2] Eyles DE, Coatney GR, Getz ME (1960). *Vivax*-type malaria parasite of macaques transmissible to man. Science.

[B3] Sandosham AA (1967). Recent researches on malaria at the Institute for Medical Research, Kuala Lumpur. Med J Malaya.

[B4] Wharton RH, Eyles DE (1961). *Anopheles hackeri*, a vector of *Plasmodium knowlesi *in Malaya. Science.

[B5] Wharton RH, Eyles DE, Moorhouse DE (1962). *Anopheles leucosphyrus *identified as a vector of monkey malaria in Malaya. Science.

[B6] Eyles DE, Warren McW, Guinn E, Wharton RH, Ramachandran CP (1963). Identification of *Anopheles balabacensis introlatus *as a vector of monkey malaria in Malaya. Bull World Health Organ.

[B7] Cheong WH, Warren McW, Omar AH, Mahadevan S (1965). *Anopheles balabacensis balabacensis *identified as a vector of simian malaria in Malaysia. Science.

[B8] Chin W, Contacos PG, Coatney RG, Kimbal HR (1965). A naturally acquired quotidian-type malaria in man transferable to monkeys. Science.

[B9] Yap LF, Cadigan FC, Coatney GR (1971). A presumptive case of naturally occurring *Plasmodium knowlesi *malaria in man in Malaysia. Trans R Soc Trop Med Hyg.

[B10] Garnham PCC (1966). Malaria parasites and other Haemosporidia.

[B11] Sandosham AA, Eyles DE, Yap LF (1962). *Plasmodium cynomolgi bastianellii *from a Malayan monkey and its similarity to aberrant *P. vivax *in man. Med J Malaya.

[B12] Warren McW, Cheong WH, fredericks HK, Coatney GR (1970). Cycles of jungle malaria in West Malaysia. Am J Trop Med Hyg.

[B13] Singh B, Lee KS, Matusop A, Radhakrishnan A, Shamsul SSG, Cox-Singh J, Thomas A, Conway DJ (2004). A large focus of naturally acquired *Plasmodium knowlesi *infections in human beings. Lancet.

[B14] Eyles DE, Laing ABG, Dobrovolny CG (1962). The malaria parasites of the pig-tailed macaque, Macaca nemstrina (Linnaeus), in Malaya. Ind J Malar.

[B15] Eyles DE, Laing ABG, Warren M, Sandoshan AA (1962). Malaria parasites of the Malayan leaf monkeys of the genus *Presbytis*. Med J Malaya.

[B16] Eyles DE (1963). The species of simian malaria: taxonomy, morphology, lifecycle and geographical distribution of the monkey species. J Parasit.

[B17] Colless DH (1956). The *Anopheles leucosphyrus *group. Trans R Entomol Soc Lond.

[B18] Chang MS, Doraisingam P, Hardin S, Nagum N (1995). Malaria and filariasis transmission in a village/forest setting in Baram district, Sarawak, Malaysia. J Trop Med Hyg.

[B19] Chang MS, Hii J, Buttner P, Mansoor F (1997). Changes in abundance and behaviour of vector mosquitoes induced by land use during development of an oil palm plantation in Sarawak. Trans R Soc Trop Med Hyg.

[B20] Chang MS, Asmad M, Fam KS (1999). Differences in *Anopheles *composition and malaria transmission in the village settlements and cultivated farming zone in Sarawak, Malaysia. Southeast Asian J Trop Med Pub Health.

[B21] Reid JA (1968). Anopheline mosquitoes of Malaya and Borneo. Stud Inst Med Res Malaysia.

[B22] Vythilingam I, Tan CH, Asmad M, Chan ST, Lee KS, Singh B (2006). Natural transmission of *Plasmodium knowlesi *to humans by *Anopheles latens *in Sarawak, Malaysia. Trans R Soc Med Hyg.

[B23] Vythilingam I, Foo LC, Chiang GL, Chan ST, Eng KL, Mahadevan S, Mak JW, Inder Singh K (1995). The impact of permethrin impregnated bednets on the malaria vector *Anopheles maculatus *(Diptera:Culicidae) in aboriginal villages of Pos Betau Pahang, Malaysia. Southeast Asian J Trop Med Pub Health.

[B24] Wharton RH, Eyles DE, Warren McW (1963). The development of methods for trapping the vectors of monkey malaria. Ann Trop Med Parasit.

[B25] Vythilingam I, Jeffery J, Harbach RE, Chan ST, Tan CH, Asmad M (2007). *Anopheles *(*Cellia*) *kokhani *N. sp (Diptera: Culicidae) from Kapit, Sarawak, East Malaysia. Proc Entomol Soc Wash.

[B26] Trung HD, Van Bortel W, Sochantha T, Keokkenchanh K, Quang NT, Cong LD, Coosemans M (2004). Malaria transmission and major vectors in different geographical areas of Southeast Asia. Trop Med Int Health.

[B27] Vythilingam I, Chan ST, Shanmugaratnam C, Tanrung H, Chooi KH (2005). The impact of development and malaria control activities on its vectors in the Kinabatangan area of Sabah, East Malaysia. Acta Trop.

[B28] Davidson G (1954). Estimation of survival rate of anopheline mosquitoes in nature. Nature.

[B29] Garret-Jones C, Grab B (1964). The assessment of insecticidal impact on the malaria mosquito's vectorial capacity from data on the proportion of parous females. Bull World Health Organ.

[B30] Garret-Jones C, Shidrawi GR (1969). Malaria vectorial capacity of a population of *Anopheles gambiae*, an exercise in epidemiological entomology. Bull World Health Organ.

[B31] Coatney GR, Collins WE, Contacos PG (1971). The primate malarias.

[B32] Warren McW, Wharton RH (1963). The vectors of simian malaria: identity, biology and geographical distribution. J Parasit.

[B33] Sallum MAM, Peyton EL, Wilkerson RC (2005). Six new species of the *Anopheles leucosphyrus *group, reinterpretation of *An. elegans *and vector implications. Med Vet Entomol.

[B34] Olumese P (2005). Epidemiology and surveillance: changing the global picture of malaria – myth or reality?. Acta Trop.

[B35] Wharton RH, Eyles DE, Warren McW, Cheong WH (1964). Studies to determine the vectors of monkey malaria in Malaya. Ann Trop Med Parasit.

[B36] Reid JA, Weitz B (1961). Anopheline mosquitoes as vectors of animal malaria in Malaya. Ann Trop Med Parasit.

[B37] Jongwutiwes S, Putaporntip C, Iwasaki T, Sata T, Kanbara H (2004). Naturally acquired *Plasmodium knowlesi *malaria in human, Thailand. Emerg Infect Dis.

[B38] Zhu HM, Li J, Zheng H (2006). Human natural infection of *Plasmodium knowlesi*. Zhongguno Ji Sheng Chong Xue Yu Ji Sheng Chong Bing Za Zhi.

[B39] Cox-Singh J, Davis TME, Lee KS, Shamsul SSG, Matusop A, Ratnam S, Rahman HA, Conway DJ, Singh B (2007). *Plasmodium knowlesi *malaria in humans is widely distributed and potentially life-threatening. Clinical Infect Dis.

